# The Antioxidant Effect of Natural Antimicrobials in Shrimp Primary Intestinal Cells Infected with *Nematopsis messor*

**DOI:** 10.3390/antiox11050974

**Published:** 2022-05-15

**Authors:** Igori Balta, Lavinia Stef, Eugenia Butucel, Gratiela Gradisteanu Pircalabioru, Adelina Venig, Patrick Ward, Myriam Deshaies, Ioan Pet, Ducu Stef, Osman Y. Koyun, Todd R. Callaway, Ozan Gundogdu, Nicolae Corcionivoschi

**Affiliations:** 1 Bacteriology Branch, Veterinary Sciences Division, Agri-Food and Biosciences Institute, Belfast BT4 3SD, UK; igori.balta@gmail.com (I.B.); eugenia.butucel@afbini.gov.uk (E.B.); 2 Faculty of Animal Science and Biotechnologies, University of Agricultural Sciences and Veterinary Medicine, 400372 Cluj-Napoca, Romania; 3 Faculty of Bioengineering of Animal Resources, Banat University of Agricultural Sciences and Veterinary Medicine—King Michael I of Romania, 300645 Timisoara, Romania; ioanpet@eurofins.com; 4 Research Institute of University of Bucharest, 300645 Bucharest, Romania; gratiela.gradisteanu@icub.unibuc.ro; 5 Faculty of Engineering and Management, University of Oradea, 410087 Oradea, Romania; adelina_venig@yahoo.com; 6 Auranta, Nova UCD, Belfield, D04 V2P1 Dublin, Ireland; pat@auranta.ie (P.W.); myriam@auranta.ie (M.D.); 7 Faculty of Food Processing Technologies, Banat University of Agricultural Sciences and Veterinary Medicine, King Michael I of Romania, 300645 Timisoara, Romania; ducustef@usab-tm.ro; 8 Department of Animal and Dairy Science, University of Georgia, Athens, GA 30602, USA; oyk52576@uga.edu (O.Y.K.); todd.callaway@uga.edu (T.R.C.); 9 Department of Infection Biology, Faculty of Infectious and Tropical Diseases, London School of Hygiene and Tropical Medicine, Keppel Street, London WC1E 7HT, UK; ozan.gundogdu@lshtm.ac.uk

**Keywords:** natural antimicrobials, antioxidant capacity, *Nematopsis messor*, intestinal primary cells, hydrogen peroxide

## Abstract

*Nematopsis messor* infections severely impact on shrimp’s health with devastating economic consequences on shrimp farming. In a shrimp primary intestinal cells (SGP) model of infection, a sub-inhibitory concentration (0.5%) of natural antimicrobials (Aq) was able to reduce the ability of *N. messor* to infect (*p* < 0.0001). To prevent *N. messor* infection of SGP cells, Aq inhibits host actin polymerization and restores tight junction integrity (TEER) and the expression of Zo-1 and occluding. The oxidative burst, caused by *N. messor* infection, is attenuated by Aq through the inhibition of NADPH-produced H_2_O_2_. Simultaneous to the reduction in H_2_O_2_ released, the activity of catalase (CAT) and superoxide dismutase (SOD) were also significantly increase (*p <* 0.0001). The antimicrobial mixture inactivates the ERK signal transduction pathway by tyrosine dephosphorylation and reduces the expression of DCR2, ALF-A, and ALF-C antimicrobial peptides. The observed in vitro results were also translated in vivo, whereby the use of a shrimp challenge test, we show that in *N. messor* infected shrimp the mortality rate was 68% compared to the Aq-treated group where the mortality rate was maintained at 14%. The significant increase in CAT and SOD activity in treated and infected shrimp suggested an in vivo antioxidant role for Aq. In conclusion, our study shows that Aq can efficiently reduce *N. messor* colonization of shrimp’s intestinal cells in vitro and in vivo and the oxidative induced cellular damage, repairs epithelial integrity, and enhances gut immunity.

## 1. Introduction

*Nematopsis messor* (*N. messor*) is a member of the Apicomplexan group of gregarines [1], commonly recognized by its spherical shape, milky white color, and even width [2]. *N. messor* is one of the main infectious agents [3] of shrimp, responsible for reduced growth rates, yellow appearance of gut tissue, and hyperplasia of the midgut epithelium [4]. These negative effects on shrimp’s health and performance prompted the shrimp industry to demand novel and innovative interventions, preferably to include natural alternatives as replacements of antibiotics. Unfortunately, these interventions are still underdeveloped, and to increase their acceptance at the farm level, further studies are necessary to decipher the biological mechanisms controlling their inhibitory effects [5].

Natural antimicrobials are usually described, both in vivo and in vitro, as efficient in preventing bacterial infections [6]. In viral and parasitic infections they have been shown to reduce the oxidative damage of the epithelium, strengthen cellular tight junctions, block the cellular pathways involved in inducing oxidative stress [7], and stimulate an immune response upon infection. All these events are mediated via the membrane toll-like receptors (TLRs) which play an essential role in the host–parasite interaction, causing epithelial tissue damage by generating an oxidative burst and proinflammatory molecules, including cytokines and reactive oxygen species [8]. Parasitic infections will also damage other membrane structures, including the Zo-1 [9] and occludin [10] proteins to achieve disruption of tight junction integrity. An example of such damage was described in the case of *Trichomonas vaginalis* infections with significant impact on cellular trans-epithelial resistance (TEER) [11]. Following infection, parasites will trigger the host NADPH oxidases to produce and release reactive oxygen species (ROS); however, eukaryotic cells are equipped with molecular mechanisms required to reduce the damaging effects of oxidation [12]. They will activate the defensive enzymes, including superoxide dismutase (SOD) [13] or catalases (CAT), to counteract the damaging effects of the oxidative burst. As part of this antioxidant response, catalases, play an important role in neutralizing free radicals [14]. Their main role will be to enable eukaryotic cells to regulate ROS production and reduce H_2_O_2_ to H_2_O [15]. Natural antimicrobials can induce changes in CAT expression upon pathogen infection, as described in bacterial infections of HEp-2 cells [16] or parasitic infections of CLEC-213 or MDBK cells [17]. In addition to blocking the molecular pathways [18] outlined above, and potentially interfere in the host–parasite interaction, they can also reduce the burden of parasite-induced oxidative stress [19].

The externally regulated kinases (ERKs) play a critical role in cellular survival during oxidative stress [20] by directly controlling the immune response through oxidative de phosphorylation [21]. In various fish species they play a vital role in regulating cell proliferation, differentiation, survival [22], and T-cell immunity [23]. Both in vitro and in vivo, it has been demonstrated that H_2_O_2_ modulates the ERK intracellular translocation with impacts on cellular proliferation and the production of immune factors [24]. Such factors include the anti-lipopolysaccharides (ALFs), which are shrimp antimicrobial peptides (AMPs) with potent antimicrobial activity against a broad range of microorganisms [25] and parasites [26]. They have been identified and classified as immune-related peptides in the hemocytes of black tiger shrimp (*Penaeus monodon*) [27]. For example, during *Vibrio harveyi* infection, the shrimp’s GIT highly upregulates the expression of AMPs of *Litvan* ALF-A, *Litvan* ALF-C, and *Lv*Dcr2, as a part of the host immune response [28].

Availability of shrimp primary cells will improve the relevance of the in vitro investigations into the inhibitory role of natural antimicrobials against *N. messor* infections of shrimp. Primary cells will retain the host tissue distinctiveness, including factors such as host genetics and disease susceptibility, efficiently replacing the in vivo experimental models [29]. Working on such cell lines is of particular importance for the development of novel interventions in shrimp farming, given that most of the current data originates from human research where primary epithelial cells provide an improved tool for studying host–parasite interactions and of other intestinal pathogens [30]. Shrimp primary intestinal cells are not yet well developed due to a lack of third party validation which caused slow progress and diminished outputs [31]. Increasing the availability of shrimp primary cells will improve the relevance of scientific data, particularly when the effect of natural antimicrobials on the host–pathogen interaction is in question [32]. Furthermore, this information will help in designing strategies for pathogen reduction in the *Penaeus vannamei* (*P. vannamei*) shrimp cultures, which represent approximately 50.3% of the total crustacean production and 6% of the total world aquaculture production [33].

Organic acids are bioactive compounds with a long history in mitigating clinical manifestations of a wide range of human and animal diseases [34]. This study was designed to better understand the cellular mechanisms targeted by natural antimicrobials during *N. messor* infection using a primary cell infection model. Specifically, we have tested the antioxidant effect of Aq on *N. messor* infection levels and its impact on tight junction resistance and the immune response. In vivo, the impact on shrimp survival was tested in a shrimp challenge test alongside CAT and SOD activity in the gut. 

## 2. Materials and Methods

### 2.1. Parasites and Antimicrobials

The gametocytes of *N. messor* were obtained, as previously described [35]. Dissected shrimps were mixed and pooled to obtain parasite culture material. On average, the number of *N. messor* per batch was 280, and this number varied among three batches (110-280). Media was supplemented with 10% concentrations of fetal bovine serum (FBS, Sigma-Aldrich, Gillingham, England, UK) and new-born calf serum (Sigma-Aldrich, Gillingham, England, UK). The media used was Dulbecco Modified Eagle Medium (DMEM) (Sigma-Aldrich, Gillingham, England, UK) supplemented with 2% penicillin–streptomycin–neomycin and 2.5 μg/mL amphotericin B as an antifungal. Forty-eight well culture plates (Thermo-Fischer, Gloucester, England, UK) were used for parasite growth in 1mL DMEM with 10% serum and loaded into a feeder-cell type pre-conditioned plate. The natural antimicrobial mixture, AuraAqua (Aq) contains 5% maltodextrin, 1% sodium chloride, 42% citric acid, 18% sodium citrate, 10% silica, 12% malic acid, 9% citrus extract, and 3% olive extract (*w/w*). The raw materials were supplied by Bio-Science Nutrition Ireland. 

### 2.2. In Vitro Infection Assay in Primary Shrimp Gut Epithelial Cells (SGP)

To prepare the primary cells (SGP), *P. vannamei* gut tissue samples were harvested. The surfaces of prawns were surface sterilized by swabbing with either 70% alcohol or 10 ppm active chlorine as bleach. Prawns were decapitated and individual tissues dissected from the prawn. SGP cells were prepared as follows. The gut was removed from the prawn and placed in a solution containing 4× penicillin 10,000 IU/mL, streptomycin 10,000 mcg/mL, fungi-zone 25 mcg/mL) and dissected into small pieces by crossed scalpel blades. The tissue fragments were washed twice with gentle centrifugation (150× *g* for 5 min). Five mL 0.25% trypsin at pH 7.4 at room temperature was added for 30–60 min and stirred on a magnetic stirrer, washed twice, and the cells were put into a 25 cm plastic culture flask with growth medium. Cultures were incubated at 28 °C. Primary cells in 24 plastic well plates (Analab, Lisburn, Northern Ireland, UK) with 0.1% DMSO (Thermo-Fischer, Gloucester, England, UK) media supplemented with 20% foetal bovine serum (FBS), 100 µg penicillin, 8% shrimp head extract, 6% salt solution, 20 ng epidermal growth factor (Sigma-Aldrich, Gillingham, England, UK), and 10 U/mL human recombinant interleukin 2 (Sigma-Aldrich, UK). Viability was measured by the trypan blue exclusion method conducted on both floating cells and attached cells. Cells excluding the dye were considered viable even if they were not proliferating. Seeded plates were incubated in laboratory temperature (RT) for 48 h prior to infection. Gametocytes of *N. messor* (80–100 gametocytes) were pre-treated for 30 min at 41°C, 5% CO_2_ with 0.5% Aq in DMEM. Secondly, primary shrimp gut epithelial cells were pre-treated with 0.5% Aq for 1 h prior to infection. A concentration of 0.5% Aq was used during each infection experiment. Gametocytes were added to primary gut epithelial cells, and at 24 h post-infection, infected monolayers were washed in 0.5 mL/well phosphate buffered saline (sodium chloride: 137 mM, phosphate buffer: 10 mM and potassium chloride: 2.7 mM) (Fisher Scientific, UK), and the number of un-attached gametocytes were counted following centrifugation of supernatant at 2000 rpm for 10 min. To study the effect of actin polymerization on H_2_O_2_ release and parasite adhesion, stock solutions of 1 mg/mL of the actin inhibitor cytochalasin D (Sigma-Aldrich, Gillingham, England, UK) (actin inhibitor) were prepared and diluted to the final concentrations using DMEM culture medium. SGP cells were incubated with 1 μg/mL cytochalasin D for 1 h at 37 °C. To inhibit the ERK signal transduction pathway, 30 µM of PD98059 (Sigma-Aldrich, Gillingham, England, UK) was added to the infection plate 60 min prior to infection. The treated cells were washed twice with PBS and infected with 80–100 *N. messor* gametocytes for 24 h. Gametocytes were counted using a Carl Zeiss inverted microscope. Counting was calculated as mean ± SD of at least four independent replicates.

### 2.3. Gene Expression Analysis

The mRNA levels of zonula occludens-1 (ZO-1) occluding expression, *Litvan* ALF-A, *Litvan* ALF-C, and *Lv* DCR2 with or without the presence ERK inhibitor PD98509 was performed, as previously described with small modifications [36]. Briefly, the exposed or infected SGP cells were snap-frozen in liquid nitrogen until use. RNA was isolated using RNeasy Plus Mini Kit (Qiagen, Manchester, England, UK). The RNA was reverse transcribed using Transcriptor First Strand cDNA Synthesis Kit (Roche, Dublin, Ireland) according to the manufacturer’s protocol. The mRNA levels were determined by quantitative RT-PCR using QuantiNovaSYBR Green PCR Kit (Qiagen, Manchester, England, UK) on a LightCycler 96 (Roche). The gene expression was normalized using the ribosomal protein lateral stalk subunit P0 (RPLP0) and glyceraldehyde-3-phosphate dehydrogenase (GAPDH). The 2–DDCT method was used to analyse the relative mRNA levels (fold changes), calculated relative to the control group.

Primers sequences are included in Table 1.

### 2.4. Transepithelial Resistance of Cellular Tight Junctions (TEER) and Actin Polymerization Assay

Transepithelial resistance measures the integrity of cellular tight junctions and is a suitable method to be used in cell culture monolayers for the purpose of measuring the effect of bacteria during infection in vitro [40]. This methodology measures the electrical resistance in ohms as a measure of cellular barrier integrity. For TEER measurement, primary cells were grown on 0.4 μm and 12 mm pore size Transwell inserts (Analab, Lisburn, Northern Ireland, UK) and were selected based on the formation of a confluent monolayer. Our aim was to investigate the effect of Aq on the barrier properties of SGP cells by taking TEER measurements at 3 h post-infection (±0.1 and 0.5% Aq) using an EVOM X meter connected to an Endohm chamber (Premier Scientific, Belfast, Northern Ireland, UK). The pyrene actin polymerization assay was performed using the actin polymerization assay kit from Abcam (kit ab239724) following the manufacturer’s protocol. Concentrations of 0.12%, 0.25%, and 0.5% of the natural antimicrobial (Aq) were tested. In a separate experiment, SGP cells were grown at 80% confluence. After treatment with 0.5% antimicrobial mixture, cells were scraped and resuspended in actin buffer containing 5mM Tris-HCl pH 8.0 and 0.2 mM CaCl_2_ and EDTA-free protease inhibitors cocktail (Roche, Dublin, Ireland); the cytosolic fraction was prepared and used (200 μg), as previously described [41]. The results were plotted in GraphPad Prism 8.

### 2.5. Challenge Tests (Counting Living Larvae)

*N. messor* was tested for its pathogenicity by a challenge test using healthy *P. vannamei* post larvae, following a procedure previously described and modified for parasitic infection [42]. Our protocol included 60 shrimp post larvae per replicate, plated in large sterile petri dishes and exposed to 80–100 gametocytes for 24 h. The antimicrobial mixture was applied at the time of infection in the concentration of 0.5% in 500 mL flasks. Survival was determined by counting the larvae at 24 h after infection. A positive and a negative control (± antimicrobial mixture or ± larvae) was also included in the challenge at 0% of the antimicrobial mixture. The experiment was performed in triplicate.

### 2.6. H_2_O_2_ Production in SGP Cells following Infection

The SGP cells were grown, as described above, until they reached confluence prior to infection with *N. messor*. The cells were routinely grown in 75 cm^2^ tissue culture flasks (Sigma-Aldrich, UK) in a humidified incubator at 37 °C with 5% CO_2_. The H_2_O_2_ production from infected and un-infected PGS cells in response to treatment with Aq was measured using the PeroxiDetect™ Kit (Sigma-Aldrich, Gillingham, England, UK), following manufacturer guidelines and the previously described procedure [43]. NADPH inhibitors including diphenyleneiodonium chloride (DPI, Sigma; 15 µM, 45 min preincubation and wash out) and bovine liver catalase (Sigma-Aldrich, Gillingham, England, UK; 300 U/mL) were used during the 24 h measuring interval.

### 2.7. Determination of Natural Antimicrobial LC_50_ Cytotoxicity and the Impact on Epithelial Cell Proliferation (MTT Assay)

The methodology used to determine the antimicrobial effectiveness against *N. messor* oocysts was performed, as previously described [17]. Parasites at a concentration of 1 × 10^7^ cells/mL in culture medium supplemented with 10% FCS were incubated for 24 h in 96-well culture dishes (Sigma-Aldrich, Gillingham, England, UK) with increasing concentrations of MT (1 to 80 µM). Experiments were performed at different Aq concentrations from 0–15% (0, 0.3, 0.5, 1, 2, 4, 10, and 15%) with all assays being performed in triplicate (*n* = 3). Additionally, cytotoxicity was also determined for each individual component of the Aq mixture (0.05–1 mg/mL). The LC_50_ was determined from oocyst survival curves (number of oocysts at each antimicrobial mixture concentration) and by identifying the concentration at which the number of oocysts was 50% that of controls. Oocyst counts were performed, as previously described [44]. Cell viability was assessed by measuring the cleavage of 3-(4,5-dimethyl-thiazol-2-yl)-2,5-diphenyl tetrazolium bromide (MTT) (Sigma-Aldrich, UK) by parasitic cells. After exposure to Aq the parasites were incubated in a solution containing 5 mg/mL MTT for 3 h at 26 °C, and the absorbance at 550 nm was read using a FLUOstar Omega plate reader (Premier Scientific, UK). To measure any antimicrobial effect on epithelial cell proliferation, the cells were cultured, as described above, and treated with a series of concentrations of Aq (0, 0.3, 0.5, 1, 4, 10, and 15%) for 24 h or treated with 0.5% Aq for 0, 3, 6, 9, 12, 15, and 24 hours to examine the dose and time-dependent impact of Aq on SGP cell viability using MTT (3-(4,5-dimethylthiazol-2-yl)-2,5-diphenyl-tetrazolium bromides) (Sigma-Aldrich, UK) assay at 37 °C. Measurements were performed in triplicate for each treatment.

### 2.8. Measurement of SOD and CAT Activity in SGP Cells and Gut Tissue

Proteins from SGP cells were prepared as a method previously described [45]. First, the cells were washed with PBS before the treatment with Trypsin PBS solution. The digested cells were centrifuged for 10 min at 1400× *g*, and the pellet was resuspended in lysis buffer containing protease inhibitors. After 30 min of incubation on ice, the extraction mixture was centrifuged at 12,000× *g* at 4 °C for 30 min, and the supernatant was transferred to a fresh tube. SOD activity was determined using a commercially available SOD colorimetric activity kit (Thermo Fisher, UK) and CAT by using a catalase activity kit (Abcam, Trumpington, England, UK, ab83464). The procedures were followed as per manufacturer instructions. For further processing, the gut tissue of challenged shrimp was disrupted by sonication for 60 s (4X) at 4 °C (in ice) in 1% saline solution followed by centrifugation at 2500 rpm at 4 °C for 5 min. The supernatant was used to determine the superoxide dismutase (SOD) and catalase (CAT) activity in *N. messor* challenged shrimp.

### 2.9. Western Blotting

SGP cells infected with *N. messor* were investigated for the inhibition of the ERK pathway following treatment with ERK inhibitor PD98509 or in the presence of Aq, as previously described [36]. Briefly, 40 μg of protein sample was separated by SDS-PAGE and transferred to nitrocellulose membranes. Membranes were blocked with 5% dried milk in Tris-buffered saline and Tween-20 (TBST, 20 mM Tris-HCl, 150 mM NaCl, 0.05% Tween-20) for 6h at room temperature and incubated with anti-phospho-ERK from Santa Cruz Biotechnology (overnight at 4 °C). After extensive washing, the membranes were then incubated with HRP-conjugated secondary antibody solution for 1 h at room temperature. The membranes were washed three times with TBST, and the blots were detected using an enhanced chemiluminescence reagent (ECL) and were exposed to photographic films (Kodak, Thermo-Fischer, Gloucester, England, UK). 

### 2.10. Statistical Analysis

Statistical analyses were performed using GraphPad software. Significance was assigned at *p*-values  <  0.05 following estimations using Student’s *t* test. Additionally, differences in dietary treatments were tested by one-way ANOVA or two-way ANOVA and when possible, with comparisons using Tukey–Kramer tests. Prior to all analyses assumptions of normality and homogeneity were tested using the Shapiro–Wilk test.

## 3. Results

### 3.1. In Vitro Cytotoxic Effects of the Antimicrobial Mixture on SGP Cells

**Figure 1:**
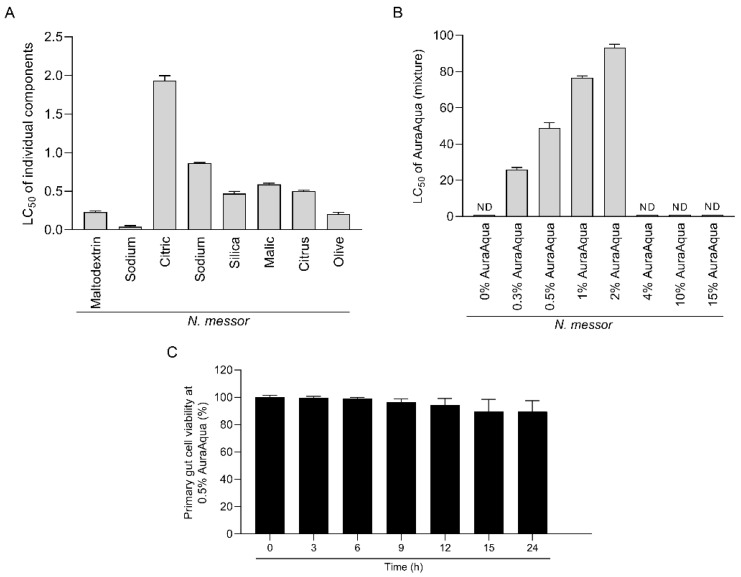
Lethal concentration (LC_50_) at which the components of Aq (**A**) or the mixture itself (**B**) reduced the number of gametocytes to half of the initial numbers. Each sample represents a mean of triplicate (*n* = 3) assays and the lethal concentration 50 (LC_50_) at which the individual components (**A**) and in mixture (**B**) reduced the number of *N. messor* gametocytes by more than 50%. The effect of 0.5% Aq on the SGP cells viability (over 24 h) is presented in panel (**C**) as determined using the MTT assay and expressed as a percentage of the untreated control cells. Values are the mean ± SE (*n* = 9). The experiments were done in triplicate and in three separate occasions.

Our first aim was to identify the cytotoxic levels of the individual Aq components, and in mixture, on the SGP cells and *N. messor*. Individually, the components decreased the number of *N. messor* gametocytes in a dose dependent manner between 0.05 and 2 mg/mL (LC_50_) (Figure 1A). When tested as a mixture (0–15%), the number of gametocytes were reduced by approximately 50% at a concentration of 0.5% (Figure 1B). The antimicrobial mixture did not affect SGP cell viability at a concentration of 0.5%, maintaining a viability of over 95% (Figure 1C). We have concluded that a sub-lethal concentration of 0.5% Aq can further be considered for in vitro and in vivo mechanistic investigations.

### 3.2. In Vitro Effect of Aq on N. messor Infection of SGP Cells

**Figure 2:**
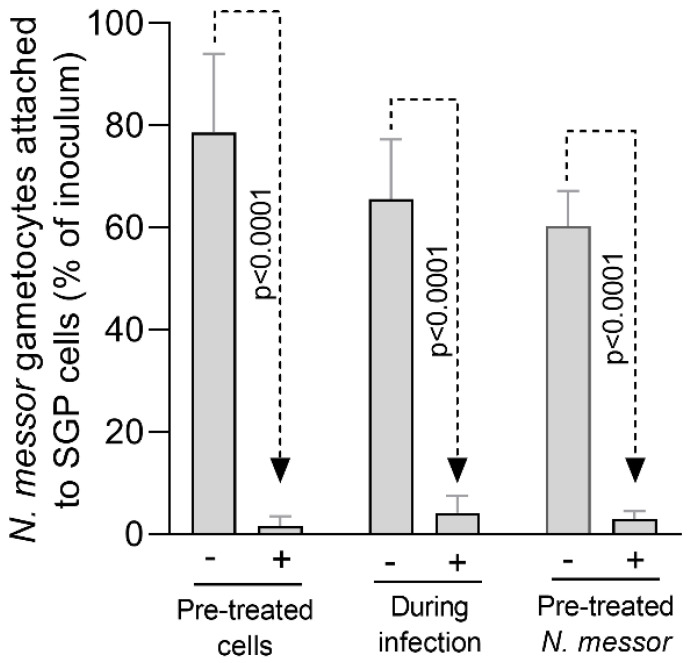
In vitro impact of 0.5% Aq on the adhesion of *N. messor* to SGP cells. Results are presented as mean ± SD of relative expressions (three biological replicates) and the *p* values are indicated on graphs.

Following the identification of the Aq sublethal concentration (0.5%) we have next investigated its effect in preventing the adhesion of *N. messor* to SGP cells. Pre-treatment of SGP cells with 0.5% Aq prior to infection, the presence of Aq during infection, or the pre-treatment of gametocytes prior to infection with 0.5% Aq significantly reduced (*p <* 0.0001) the number of N. *messor* gametocytes (Figure 2) attached to SGP cells. The dual infection inhibitor effect of Aq, on both the parasite and the host, suggested the need for further investigations to unlock the nature of its effect.

### 3.3. Natural Antimicrobials Strengthens Cellular Tight Junctions (TEER) and Prevents Actin Polymerisation during Infection

**Figure 3:**
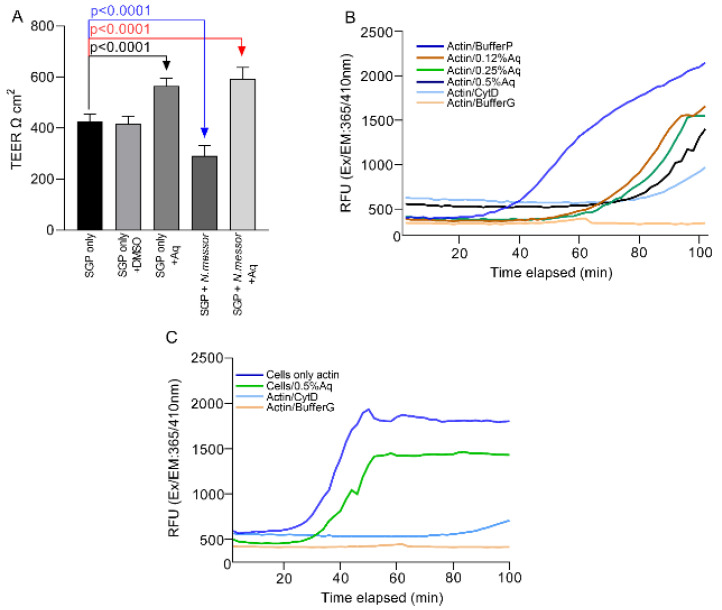
Transepithelial resistance of SGP cells infected with *Nematopsis messor* at 24 h post-infection using 0.5% Aq during infection (Panel (**A**)). In vitro actin polymerization assay (Panel (**B**)) was measured in kinetic mode fluorometer at different concentrations of Aq (0.12, 0.25, 0.5%). SGP cellular actin under 0.5% Aq is presented in Panel (**C**). Significant differences compared to uninfected cells are indicated on the graph Error bars indicate standard deviations (*n* = 9).

To understand the precise role of Aq, we next investigated its involvement in restoring TEER and preventing cellular actin polymerization during *N. messor* infection of SGP cells. Our results show that tight junction resistance (TEER) is restored in the presence of 0.5% Aq (Figure 3). At 24 h post-infection, the TEER of infected and Aq treated SGP cells increased significantly (*p* < 0.0001) compared to the untreated infected cells where a significant decrease was observed (*p* < 0.0001) (Figure 3). To further investigate the mechanism involved in reduced parasite adhesion, we further tested the ability of Aq to prevent cellular actin polymerization in vitro. Results confirmed that the highest inhibition of actin polymerization was achieved at concentrations of 0.5% Aq (Figure 3B). In a separate experiment analyzing the effect of Aq on SGP cellular actin, we observed inhibition of actin polymerization over time (Figure 3C). These results indicate that the antimicrobial mixture inhibits the disruption of the cellular tight junctions and prevents actin polymerization resulting in reduced *N. messor* attachment to SGP cells.

### 3.4. The Impact of Aq on the Induced Oxidative Stress in N. messor Infected SGP Cells 

**Figure 4:**
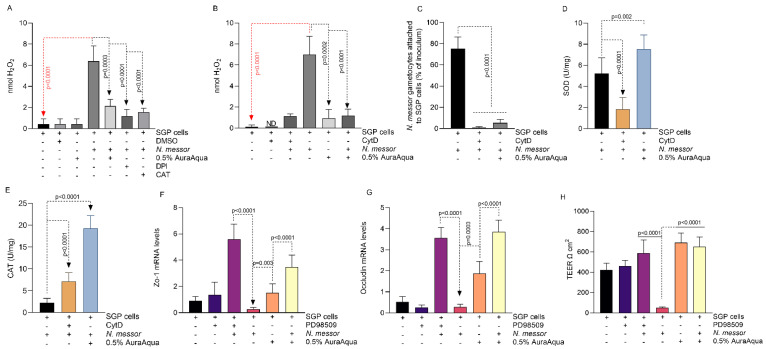
The effect of Aq on *N. messor* infected SGP cell membrane integrity. Panel (**A**) shows the H_2_O_2_ levels in *N. messor* infected SGP cells in the presence of Aq, and Panel (**B**) shows the impact of the impact of CytD cytoskeleton inhibitor. The impact on *N. messor* adhesion to SGP cells is presented in Panel (**C**). Panel (**D**) presents the SOD activity, and (**E**) presents the catalase (CAT) in Aq-treated infected cells. Zo-1 and occluding mRNA levels are shown in Panels (**F**,**G**), respectively, followed by the TEER values in Panel (**H**). Data are presented as means (SD) of three independent experiments. *p* values are indicated on graph to indicate significance.

To further investigate the role of Aq, we have assessed its ability to reduce the parasite-induced host oxidative stress. At 24 h post-infection, the levels of H_2_O_2_ released by the infected SGP cells increased significantly (*p <* 0.0001); however, a significant decrease (*p =* 0.0003) was observed in the presence of 0.5% Aq (Figure 4A). Treatment of infected cells with DPI and catalase (CAT) inhibitors directly linked Aq with the inhibition of host NADPH oxidases. Our results established that treatment of infected SGP cells with 0.5% Aq reduced the levels of host-released H_2_O_2_. Similar effects were also observed in the presence of both DPI and catalase inhibitors (*p <* 0.0001) (Figure 4A). Moreover, the inclusion of CytD or 0.5% Aq during *N. messor* infection of SGP cells also reduced H_2_O_2_ production (Figure 4B) and parasite adhesion (Figure 4C), indicating that the antioxidant effect of Aq is established by blocking cell actin polymerization and the physical interaction between *N. messor* and the SGP cells. The decrease in H_2_O_2_ release in infected and Aq-treated SGP cells was simultaneous to the increase in SOD (Figure 4D) and catalase (Figure 4E) activity.

We also hypothesized that lower H_2_O_2_ levels will inhibit the activation of the ERK-signaling pathway and prevent disruption of cell membrane integrity. First, we show that Aq repairs cell membrane integrity by increasing the mRNA levels of ZO-1 (Figure 4F) and occludin (Figure 4G) in Aq-treated infected SGP cells. Similar effects were observed when the ERK-signaling pathway was inhibited with the PD98509 inhibitor. Both the PD98509 and Aq prevented (*p <* 0.0001) TEER disruption in infected cells (Figure 4H). These results suggest that Aq blocks H_2_O_2_ activation of the ERK-signaling pathway and reduces host oxidative stress by increasing CAT and SOD activity. Moreover, we show that the physical interaction between *N. messor* and the SGP cells is required to induce oxidative stress and that such interaction can be prevented with Aq.

### 3.5. AuraAqua Inhibits the Production of Immune-Related Antimicrobial Peptides in Infected SGP Cells by Blocking the ERK-Signaling Pathway

**Figure 5:**
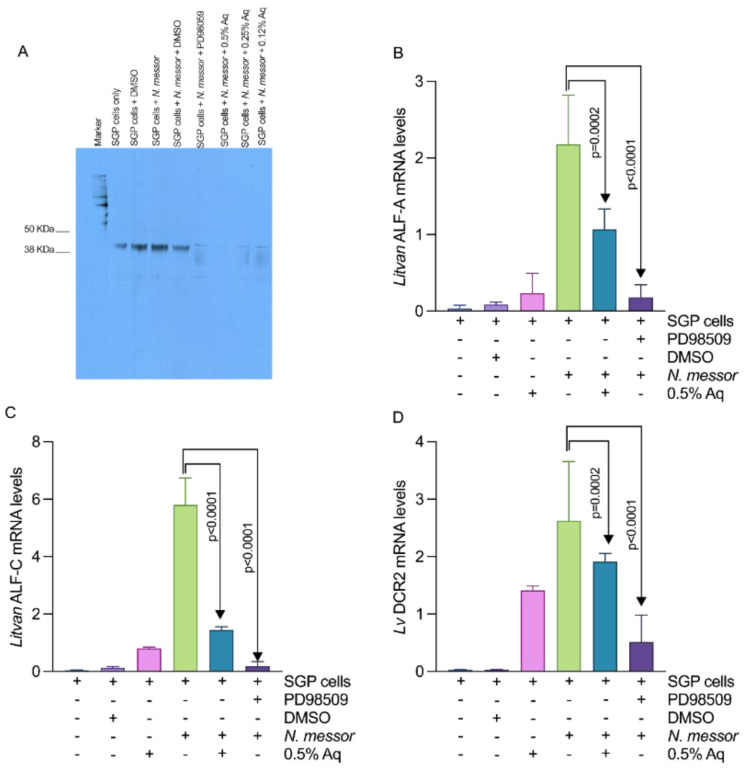
The effect of Aq on ERK tyrosine phosphorylation (Panel **A**) and the *Litvan* ALF-A (**B**), *Litvan* ALF-A (**C**), and *Lv* DCR2 (**D**) mRNA levels in *N. messor* infected SGP cells. P-Tyr levels were assessed in infected SGP cells in the presence of 0.5% Aq and of the ERK inhibitor, PD98509. Experiments were performed in triplicates. *P* values are indicated on graph to indicate significance.

The involvement of the ERK pathway in the infection process prompted us to investigate Aq involvement in regulating the activity of immune-related antimicrobial peptides known to be upregulated in damaged shrimp gut tissue as a response to parasite infection (*Litvan* ALF-A, *Litvan* ALF-C and *Lv*Dcr2) [28]. Our results showed that both Aq and the ERK inhibitor (PD98509), are inactivating the ERK pathway in *N. messor* infected SGP cells through tyrosine dephosphorylation (Figure 5A). Inactivation of the ERK pathway by either 0.5% Aq or by PD98509 significantly reduced the mRNA levels of *Litvan* ALF-A (Figure 5B), *Litvan* ALF-C (Figure 5C), and *Lv*Dcr2 (Figure 5D) in infected *N. messor* SGP cells. These results suggest that Aq reduces *N. messor* pro-inflammatory events in SGP cells through the inactivation of the ERK signal transduction pathway.

### 3.6. Challenge Tests

**Figure 6:**
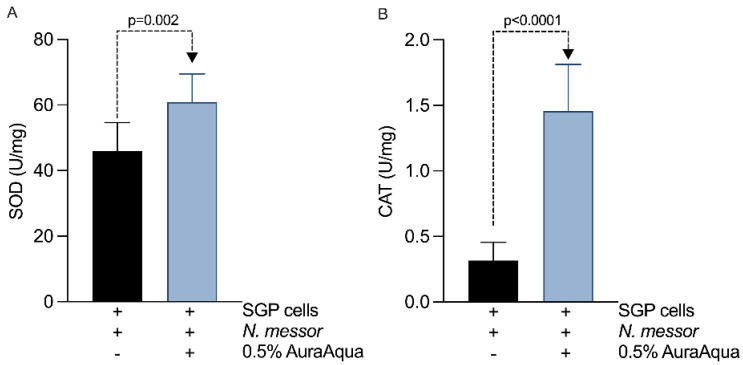
The effect Aq on the antioxidative capacity of *P vannamei*: (**A**) SOD activity, (**B**) CAT activity. Bars and error bars represent the means ± SD. Measurements were performed in triplicates. *p* values are indicated on graph to indicate significance.

We have next attempted to translate in vivo the observed in vitro antioxidant effects of Aq. To gain in vivo relevance, we have performed challenge studies by infecting *P. vannamei* shrimps with *N. messor* with or without Aq treatment. The results presented in Table 2 show that 0.5% Aq reduced to 14% the mortality rates in infected shrimp (Table 2) from a rate of 68% recorded in the infected and untreated control group compared to after 24 h of exposure. The activity of SOD (Figure 6A) was significantly increased (*p =* 0.002) in the gut tissue of the infected and treated group. Catalase followed a similar trend (Figure 6B) of increased activity (*p <* 0.0001) in the infected and treated group and was maintained to significantly lower levels in the control group. The challenge tests further confirmed the in vitro antioxidant effect of Aq and recommends its use in shrimp farming to prevent parasitic infections.

## 4. Discussion

Parasitic colonization of *P. vannamei* gastrointestinal tract (GIT) has a negative impact on shrimp productivity [46]. Therefore, controlling GIT colonization represents an important step for successful and economically viable farming of crustaceans [47]. Controlling parasitic colonization with novel and innovative interventions (e.g., plant extracts, antibiotics, recombinant vaccines, and DNA vaccines) is still widely unaccepted by farmers given the insufficient scientific evidence to support their beneficial effects [46] and modes of action [48]. Hence, most of the current therapies are antibiotic based, yet used with little success as anti-*Nematopsis* treatments [49]. Mixtures of organic acids represent an alternative to the use of antibiotics, and they have been shown to also exert an efficient antimicrobial effect [36]. In this study, *N. messor* infected shrimp primary intestinal cells were used to improve our understanding of the biological mechanisms by which mixtures of natural antimicrobials exert their anti-parasitic effects.

The release of H_2_O_2_ in the extracellular space as a result of the host–pathogen interaction and its inhibition upon exposure to natural antimicrobials was previously demonstrated in parasite [36] or viral infections [50]. Natural antimicrobials (rosmarinic acid) have been previously shown to inhibit the activity of NADPH Nox4 oxidases and reduce inflammation in HEK cells [51]. Our study extends this observation to SGP cells, which upon infection with *N. messor* triggers the production of reactive oxygen species (ROS) by the host NADPH oxidases. Furthermore, we show that Aq increases both the superoxide dismutase (SOD) and catalase (CAT) activity, causing the decrease in H_2_O_2_ detection in infected and Aq-treated SGP cells. It is unusual for SOD only to be involved in reducing the levels of H_2_O_2_; however, this process is indeed plausible [52]. Considering also the observed increase in catalase activity, we can assume that both CAT and SOD can trigger the reduced peroxide level. Since the host–parasite interaction is essential for H_2_O_2_ production and release, a process inhibited by natural antimicrobials, we have further investigated the biological mechanisms involved.

Inhibition of host actin polymerization is critical for both the host–parasite interaction [53] and H_2_O_2_ formation and release in the extracellular space [54]. The importance of actin polymerization in the *N. messor* interaction with SGP cells was proven by using the inhibitor cytochalasin D, known for being involved in the final extent of the Mg^2+^ induced polymerization process [55]. Pre-treatment of SGP cells with cytochalasin D or Aq significantly inhibited the adhesion of *N. messor* as well as H_2_O_2_ formation and release. As proven in vitro, the inhibition of adhesion indicates that Aq is involved in actin rearrangement, necessary in vivo for the efficient invasion of the host epithelium. Transepithelial resistance (TEER) is another important feature of the eukaryotic epithelium integrity which is weakened during the host–pathogen interaction [43], including parasitic infections [56]. Natural antimicrobials restore the integrity of the cellular tight junctions [57] and have the ability to reduce parasitic infections [53]. Herein, we show that TEER is reduced in *N. messor* infected SGP cells and restored in the presence of Aq. It is unclear how this mechanism unfolds in host–parasite interactions; however, as described in this study, Aq blocks H_2_O_2_ activation of the ERK-signaling pathway and restores tight junction structures [36]. The restoration of tight junction integrity and the increased TEER values is significant as it plays an important role in parasite virulence since it has been shown that other protozoa (e.g. *Giardia duodenalis*) also disrupt the intestinal barrier during invasion of epithelial cells [58].

Anti-lipopolysaccharide factors (ALFs) are antimicrobial peptides only found in marine organisms, including penaeid shrimps [38]. Transcripts of *Litvan* ALF-A, *Litvan* ALF-C were mainly found in the foregut and midgut of shrimp, especially in response to tissue damage and parasite infection [59]. Peroxidases, such as peroxinectin, also known as a cell ligand molecule in *Pacifastacus leniusculus* crayfish [60], controls the expression of antimicrobial peptide genes in the hemocytes of red swamp crayfish (*Procambarus clarkii*) [61] and was identified as being significantly reduced in black tiger shrimp (*Penaeus monodon*), mimicking a fungal infection [62]. Little is known about the expression and distribution of these immune-related genes in the gut and in hepatopancreas; however, they are reportedly involved in both antimicrobial and antiviral defenses in response to parasite and bacterial infections [28]. In viral infections, *Lv*Dcr2 contributes to the activation of anti-viral immunity in an RNAi pathway independent fashion through the regulation of other RNAi immune pathways, resulting in activation of anti-viral immunity [39]. Parasites activate the externally regulated kinases, ERKs, and when inhibited by the inhibitor PD98509, reduces proinflammatory cytokine production [63]. Both Aq and PD98509 were able to significantly downregulate in the expression of *Litvan* ALF-A, *Litvan* ALF-C, and *Lv*Dcr2 in *N. messor* infected SGP cells, suggesting their involvement in restoring gut health in response to ERK-mediated *N messor* tissue damage. The ability of natural antimicrobials (Quzhou Fructus Aurantii) to attenuate ERK phosphorylation and reduce the levels of pro-inflammatory cytokines was also confirmed both in vivo and in vitro in LPS-induced lung injury and RAW 264.7 cells [64]. Similar results were observed with *Spirulina maxima* extracts that reduced ERK phosphorylation and reduced ROS formation and LPS-induced inflammation in RAW264.7 cells [65].

Further investigation into how natural antimicrobials prevent the devastating effects of *N. messor* infections will help shrimp producers to efficiently establish and implement the most appropriate interventions at the farm level. These types of approaches were proven efficient in finding a solution for a *Vibrio* outbreak at Ecuadorian hatcheries where scientific investigations helped in selecting the three of the most efficient natural products out of 16 in controlling the disease [42]. Blends of organic acids are also efficient in improving nutrient utilization in diseased shrimp (e.g. *Vibrio* infections), indicating a better resistance to bacterial infections and higher survival rates [66]. The current study supports these findings, indicating that mixtures of natural antimicrobials (Aq) improved the survival rates and reduced oxidative events in the GIT of shrimps infected with *N. messor*. The outcomes of this study could potentially contribute to the general efforts to reduce the use of prophylactic antibiotics in aquaculture and replace them with novel alternatives which are less detrimental to the fisheries industry and to the environment overall.

## 5. Conclusions

Mixtures of natural antimicrobials aim to reduce or prevent parasitic infections in shrimp to enhance health and maintain and improve on farm performance. Our study suggests that Aq can reduce *N. messor* pathogenicity both in vitro and in vivo by preventing cellular actin polymerization and by restoring the gut cell membrane integrity. The in vitro oxidative stress induced by *N. messor* adhesion to SGP cells is significantly reduced by the antimicrobial mixture by increasing the activity of SOD and CAT, resulting in reduced H_2_O_2_ production. The decreased levels of H_2_O_2_ prevented the activation of the ERK signal transduction pathway and reduced the expression of antimicrobial peptides usually produced in damaged tissue in the foregut and midgut of parasite-infected shrimps. In conclusion, this study further clarifies the anti-parasitic and anti-oxidative mechanisms of natural antimicrobials with an emphasis on the beneficial host effects.

## Figures and Tables

**Table 1:** Primer sequences.

Gene Name	Gene Accession Numbers	Primer Sequence Forward/Reverse (5′-3′)	Reference
SOD	XM_027376215.1	(F) GCAATGAATGCCCTTCTACC(R) CAGAGCCTTTCACTCCAACG	[37]
*Litvan* ALF-A	EW713395	(F) CTGATTGCTCTTGTGCCACG(R) TGACCCATGAACTCCACCTC	[38]
*Litvan* ALF-C	FE058235	(F) ATGCGAGTGTCTGTCCTCAG(R) TGAGTTTGTTCGCGATGGCC	[38]
*Lv* DCR2	HQ541163	(F) AGGAAATGCAATGTCGTGGTT(R) ACGAGCCCTCCCCCTAGATT	[39]
*Zo-1*	NM_0013011025.3	(F) CGGGACTGTTGGTATTGGCTAGA(R) GGCCAGGGCCATAGTAAAGTTTG.	[36]
*Occludin*	NM_001205254.2	(F) TCCTATAAATCCACGCCGGTTC(R) CTCAAAGTTACCACCGCTGCTG.	[36]

**Table 2:** Mortality of *P. vannamei* after 24 h of challenge with *N. messor*.

Mortality	**Aq (%)**	
	0	0.5
	68 ± 0.45	14 ± 1.22

## Data Availability

Data is contained within the article.
